# Supplementary material for: Are carbon emissions trading and green financial instruments synergistic? -Comprehensive quantitative research based on content analysis
Source: PLoS One. 2024 Mar 7;19(3):e0298601. doi: 10.1371/journal.pone.0298601 (PMC10919601; doi:10.1371/journal.pone.0298601)
Supplement: S1 Data — (ZIP) [file pone.0298601.s001.zip › Data/地级市Z_F工作报告.zip!/╕ú╜¿/┴·╤╥╩╨/└┤╫╘╣1⁄2╓┌║┼í╛┬φ┐╦╩2╛▌═°í┐_2007.docx]

　—2007年3月26日在龙岩市第三届人民代表大会第一次会议上的报告

　　龙岩市人民政府市长 雷春美

各位代表：

　　现在，我代表市人民政府向大会作政府工作报告，请予审议，并请市政协各位委员和其他列席人员提出意见。

　　阔步前进的五年

　　2002年市二届人大一次会议以来，我市各级政府在省委、省政府和市委的正确领导下，坚持以邓小平理论和“三个代表”重要思想为指导，全面落实科学发展观，努力构建和谐社会，深入贯彻党的十六大、十六届三中、四中、五中、六中全会和胡锦涛总书记、温家宝总理视察我市时的重要指示以及省第八次党代会、市第三次党代会精神，扎实推进生态型经济枢纽和海峡西岸经济区重要增长极建设。经过全市人民的共同努力，顺利完成了市二届人大一次会议确定的各项目标任务。

　　五年来，我们的主要工作和成效是：

　　1、经济实力持续增强，发展质量稳步提升。2006年与2001年相比，地区生产总值由222亿元增加到448.6亿元、年均增长10.9%，增速从全省第九位上升到第五位；全社会固定资产投资由54.3亿元增加到164.9亿元，累计514.8亿元，超过1978—2001年的总和；社会消费品零售总额由80.1亿元增加到145.9亿元，年均增长12.7%；财政总收入由34.1亿元增加到81亿元，其中地方级财政收入由13.3亿元增加到28.5亿元，年均分别增长18.9%和16.5%；万元GDP能耗由1.91吨标准煤下降到1.43吨标准煤，下降了25.1%。规模以上工业经济效益综合指数从2004年4月起连续35个月居全省首位。

　　2、“三农”工作持续加强，新农村建设步伐加快。认真落实各项支农惠农政策，启动“十镇百村”新农村建设试点，建立农村“六大员”队伍，市级投入支农资金4.5亿元。农村经济稳步发展，农林牧渔业总产值年均增长5.6%，粮食生产保持稳定，农业五大主导产业和五大特色产品更具竞争力，共有32个农产品获绿色食品标志使用权、85个农产品获无公害食品认证。农业产业化步伐加快，现有市级以上龙头企业71家和农民专业合作组织、农产品行业协会180个。完成农村劳动力技能培训8.9万人，累计转移就业76.4万人。农村“六通”工程建设进展顺利，实现100%的村通电、84%的村通村道路路面硬化、64%的村通客车、99%的村通固定电话，91%的村实施了人饮工程，80%的村能收看市、县电视节目；水利“六千”工程、林业“八大”工程和农业综合开发、商品粮基地、烟田基础设施、土地整理、户用沼气等工程建设扎实推进；防灾减灾体系进一步健全，基层防汛、防台风预警应急体系建设得到国家防汛抗旱总指挥部的肯定。与台湾的农业合作得到加强。小城镇和新村规划建设稳步推进，农村环境卫生整治力度加大，人居环境得到较大改善。

　　3、产业结构持续升级，工业总量明显壮大。三次产业结构由2001年的25.5:37.2:37.3调整为19.5:47.3:33.2。工业总产值由217亿元增加到483.5亿元，年均增长16.4%；工业增加值占GDP的比重达41.8%，提高10.3个百分点；烟草、机械、钢铁、建材、纺织、电力、煤炭、农产品加工等重点产业对规模以上工业增长的贡献率达到80.4%；新增规模以上工业企业466家、累计809家，产值亿元以上工业企业41家、累计60家，上市公司3家、累计4家，省级高新技术企业由3家增加到12家。2005—2006年累计获批工业用地2.6万亩，相当于前12年的总和；2005年下半年以来累计建成标准厂房228万平方米；8个省级工业园区和首批15个乡镇工业集中区建设加快。现有中国驰名商标4件、省著名商标29件，中国名牌产品4个、省名牌产品37个，国家免检产品7个，国家地理标志保护产品1个。旅游、商贸、金融、保险、房地产等服务业快速发展，建成全省新兴旅游大市，闽粤赣边重要物资集散地的作用日益显现。

　　4、基础设施持续改善，投资环境日益优化。投入公路、铁路建设资金153.7亿元。龙厦高速公路、赣龙铁路、冠豸山机场相继建成，龙岩至北京“海西号”快速旅客列车顺利开行，龙长高速公路建设加快推进，龙厦铁路、永武高速公路、古田大道开工建设。完成国、省道及市通县公路路面改造560公里，农村公路路面水泥硬化5600公里，县通乡镇道路路面全部实现硬化。新增发电能力94.6万千瓦，电力装机容量达272万千瓦；220千伏输变电站由2座增加到4座。移动电话用户、互联网用户分别由75万户、9万户增加到110万户和16.9万户。投资1.45亿元完成166家大气污染企业治理，关闭了中心城市5家水泥污染企业，中心城市环境空气质量良以上天数由2001年的185天增加到287天；九龙江、汀江、闽江流域水环境污染综合整治扎实推进。出台了《关于促进投资与企业发展的若干意见》等一系列优惠政策。组建了行政服务中心和项目中心，取消了58.1%的市级行政审批事项，推行告知承诺、代办审批、网络审批等服务措施，市级审批事项立即办理率达78.5%。通关环境不断改善。制定了《关于对损害经济发展软环境行为实行效能告诫的暂行规定》，建立了政府及其部门绩效评估制度和市直部门投资与发展环境监测评估制度，各级各部门服务发展力度持续加大。根据省外资工作领导小组牵头组织的投资环境测评调查，我市投资环境测评结果从2002年的全省第八位上升到2005年的第二位。

　　5、中心城市持续拓展，带动能力逐步增强。城市规划体系基本形成，总规修编完成《纲要》编制，控规覆盖率达60平方公里。组织实施了2003—2007年中心城市百个城建项目建设，市级财政完成投资15.8亿元。新建50亩以上住宅小区11个、主次干道21条、15层以上中高层建筑18座、市政公用设施24项，新建城市道路76公里、排污管道90公里、垃圾无害化处理场1座、公共绿地248.6万平方米，自来水普及率达100%，污水处理率达68%，垃圾无害化处理率达95.3%，人均公共绿地达8.5平方米。龙岩经济开发区、龙州工业园区等城市新区初具规模，中心城市建成区面积由26.5平方公里扩大到31.6平方公里。中心城市管理力度加大，城市美化、亮化、绿化成效明显，获省级园林城市和省级卫生城市称号。

　　6、对外开放持续扩大，各项改革扎实推进。登记注册外商投资企业224家、注册资金5.1亿美元、外方认缴资金4.5亿美元，比前五年分别增长2.5倍、5.2倍和5.5倍；外贸出口总额6.7亿美元,新增152家企业获外贸进出口经营权；内联引资合同利用市外资金362.2亿元，实际到资139.3亿元。截止2006年底，全市登记注册资本100万元以上的私营企业2339户，其中注册资本1000万元以上394户，分别比2001年底增长4.9倍和18.7倍。“11&#8226;18”中国龙岩投资项目洽谈会已成功举办三届，成为我市扩大招商引资和推进对外经贸合作交流的重要平台。与友好城市澳大利亚伍龙岗市和世界各地客属组织的联系不断加强。市、县、乡党政机构改革和事业单位改革稳步推进。完成206家国有工业企业改制，置换职工身份4.7万人，企业改制面和职工身份置换面分别达到95.8%和97.5%。成立了市国有资产监督管理委员会，建立了国有资产管理营运体系。农村税费改革全面推行，免征农业税及其附加和除烟叶外的农业特产税及其附加1.5亿元。粮食购销市场化、集体林权制度、小型水利设施产权制度等改革不断深化，农村工作机制创新取得新进展。投资体制、公共财政、社会事业、城市公用事业等改革向纵深推进。

　　7、社会事业持续发展，群众生活水平不断提高。获国家、省科技进步奖24项，创建省级以上企业技术中心6家，其中龙净环保和紫金矿业公司跻身全国首批118家国家级企业技术中心，启动市科技创业园建设。创办了龙岩学院，组建了闽西职业技术学院；职业教育稳步发展；高考成绩连续五年居全省前列，新罗、永定“双高普九”通过省级验收；全面落实农村贫困家庭子女义务教育阶段“两免一补”政策，对农村义务教育阶段学生全部免收学杂费，完成33万平方米农村中小学D级危房改造；市、区教育资源得到有效整合。组建了市疾病预防控制中心、卫生监督所和传染病院；新建市第一医院病房大楼、中医院门诊大楼等一批卫生基础设施，实施了乡镇卫生院“百院建设、千人培训”计划；在新罗、永定、上杭、长汀4县（区）开展了新型农村合作医疗和农村医疗救助试点，启动了新罗区城市医疗救助试点；卫生、食品、药品监督管理不断加强，成功抵御“非典”和高致病性禽流感等重大疫情。新建了龙岩图书馆、体育公园、体育大厦等文体设施，完成了古田会议旧址群维修保护一、二期工程；永定客家土楼入选“世遗”预备名单；我市体育健儿参加奥运会实现金牌零的突破，参加第十三届省运会共获金牌116枚、金牌总数超过前12届总和、居全省第四位。国土资源科学开发与规划保护工作进一步加强，连续7年实现耕地占补平衡。连续16年完成省下达的人口控制计划。城镇登记失业率由6.4%下降到4.7%。养老、失业、医疗、工伤、生育“五大保险”稳步扩面，城乡低保基本实现应保尽保，增加了企业退休人员基本养老金，在我省率先实施了农民房屋保险并在全省推广，扶贫开发、社会救助和慈善事业得到加强。偿还建设领域拖欠工程款3.8亿元，清付农民工工资3553万元。完成莲东小区等一批经济适用房建设，城镇居民人均居住面积由24平方米扩大到32平方米。实施为民办实事项目71项，解决了一批关系群众切身利益的实际问题。与2001年相比，城镇居民人均可支配收入由6579元增加到11718元，年均增长12.2%；农民人均纯收入由3078元增加到4492元，年均增长7.9%。

　　8、民主法制建设力度持续加大，精神文明建设继续深化。认真执行人大及其常委会的决议决定，自觉接受人大及其常委会的法律监督、工作监督，支持政协履行政治协商、民主监督和参政议政职能，加强与民主党派、工商联、无党派人士和各人民团体的联系，虚心听取各界人士的意见。市二届人大一次会议和市政协二届一次会议以来，交由政府系统承办的763件人大代表建议和1351件政协提案已全部办结，人大代表和政协委员满意率分别达到92.3%和96.7%。全面推进依法行政，推行行政执法责任制，强化行政执法监督，认真办理行政复议案件，加强规范性文件管理。“四五”普法通过省级验收，“五五”普法顺利推进。廉政建设、政务公开、审计监督等工作进一步加强。“平安龙岩”建设和社会治安综合治理成效显著，社会治安满意率达94.4%，被中央综治委授予“2001—2004年度全国社会治安综合治理优秀地市”称号。应急管理工作得到加强。社区建设稳步推进。全面落实安全生产责任制，安全生产形势逐年好转。积极推进富有闽西特色的精神文明建设，群众性精神文明创建活动不断深化，获“全国双拥模范城”和创建省级文明城市工作先进城市称号。武平、漳平被中央党史研究室确认为原中央苏区范围。国防动员、民兵预备役、人民防空等工作取得新进展。统计、民族宗教、外事侨务、老区、信访、档案、地方志、老年人、妇女儿童、残疾人等工作进一步加强。

　　2006年，是龙岩发展历史上不平凡的一年。胡锦涛总书记、温家宝总理视察我市，充分肯定了龙岩老区的发展变化和各项工作，并寄予厚望，体现了党中央、国务院对龙岩发展的极大关心和支持，给全市人民以巨大鼓舞。一年来，全市上下认真贯彻落实总书记和总理的重要指示，把老区建设与海西建设结合起来，同心协力，艰苦奋斗，攻坚克难，开拓进取，战胜了历史罕见的自然灾害，经济社会发展取得了明显成效。地区生产总值增长12.5%，是1998年以来增速最快的年份。财政总收入突破80亿元大关，增长32.6%，其中地方级财政收入28.5亿元，增长36.8%。全社会固定资产投资增长38.4%。社会消费品零售总额增长13.4%。实际利用外资增长54%。内联引资实际利用市外资金增长41.3%。城镇居民人均可支配收入增长10.2%，农民人均纯收入增长8.9%。各项改革不断深化，社会事业稳步发展。成功承办和举办了全省纪念红军长征胜利70周年大会、第十三届省运会、第七届省老运会、第三届中国龙岩投资项目洽谈会等一系列重大活动。这些成绩的取得，保证本届政府顺利完成了市二届人大一次会议确定的各项目标任务。

　　各位代表，五年的成就令人鼓舞，五年的历程非同寻常。这是在省委、省政府和市委的正确领导下，在市人大及其常委会的法律监督、工作监督和市政协的民主监督下，全市人民团结一心、奋力拼搏的结果。在此，我代表市人民政府，向为经济社会发展付出辛勤劳动的全市人民，向给予政府工作大力支持与有效监督的人大代表、政协委员和民主党派、工商联、无党派人士、各人民团体、离退休老同志，向为龙岩改革发展稳定作出积极贡献的中央和省驻岩机构、驻岩部队、武警消防官兵、公安政法干警，向所有关心支持龙岩发展的港澳台同胞、海外侨胞、国际友人和各界人士，表示衷心的感谢！

　　回顾五年的工作，我们深切地体会到：必须坚持科学发展，努力实现又好又快。全面贯彻落实科学发展观，正确处理“好”与“快”的关系，突出持续与提升，突出协调与和谐，做到“好”字当头，“快”在其中，努力实现速度、质量、效益相协调，消费、投资、出口相协调，人口、资源、环境相协调。必须坚持围绕大局，牢牢把握发展机遇。立足海西、服务全局，主动对接、奋勇争先，紧紧抓住国家鼓励东部地区率先发展、支持海西建设、支持老区发展三大历史机遇，充分发挥生态、资源和对内联接三大优势，当好前锋，多做贡献，在服务海西建设中积极作为。必须坚持项目带动，不断拓展发展空间。坚持不懈地实施项目带动战略，不断深化“二三四五”项目工作思路，推进“三个一百”、重点项目建设计划和“双百”项目竞赛活动，以项目集聚要素、扩大投资、改进服务、推动发展。必须坚持以人为本，加快构建和谐社会。把实现好、维护好、发展好最广大人民群众的根本利益作为一切工作的出发点和落脚点，把促进社会发展与解决民生问题摆在更加突出的位置，努力做到发展为了人民，发展依靠人民，发展成果由人民共享。必须坚持为民负责，加强政府自身建设。努力建设对人民高度负责的政府，把大力弘扬老区精神与新时期创业精神结合起来，增强行政能力，提高服务效能，创新管理方式，加强廉政建设，树立良好形象，凝聚方方面面的力量推进发展。

　　五年来，我们取得的成绩是显著的，但是经济社会发展中仍然存在不少困难和问题，主要是：经济增长速度低于全省平均水平，经济总量尤其是工业总量仍然偏小，大企业和大项目不多；经济增长方式尚未根本转变，非资源型产业比重偏小，能源消耗偏高，生态保护和环境污染治理仍需加强；城乡、区域之间发展不平衡，农民持续增收难度大，农村发展仍然滞后，城市管理长效机制有待进一步健全；劳动者技能素质与岗位需求的矛盾比较突出，社会保障体系不够健全，部分群众生活比较困难；影响社会安定稳定的因素仍然存在；一些公务员的思想素质、行政能力和工作作风与新形势、新任务的要求还不完全适应，有的地方形式主义、官僚主义和消极腐败现象仍然存在。对这些问题，我们将认真对待，采取更加有力的措施，切实加以解决。

　　又好又快地推进龙岩新一轮大发展

　　今后五年，是推进科学发展，促进社会和谐，实现龙岩老区更好更快发展的关键时期，我们要完成“十一五”规划，制订和实施“十二五”规划，在海峡西岸经济区建设中争取更大作为。今后五年政府工作的指导思想是：以邓小平理论和“三个代表”重要思想为指导，牢固树立和落实科学发展观，加快构建和谐社会，认真贯彻党的十六大以来的各项方针政策和胡锦涛总书记、温家宝总理视察我市时的重要指示精神，围绕省委、省政府建设海峡西岸经济区的总体部署和我市建设生态型经济枢纽、成为海峡西岸经济区重要增长极的目标定位，落实“四个重在”的实践要领、“前、通、借、联”的发展要求和市第三次党代会的部署，全面推进社会主义经济建设、政治建设、文化建设和社会建设，又好又快地推进新一轮大发展，充分发挥龙岩在海西纵深推进中的前锋作用。

　　主要目标是：到2011年，地区生产总值年均增长11%以上，财政总收入年均增长13%以上，工业总产值年均增长16%以上，万元GDP能耗比2006年下降15%，环境质量按功能分区达到国家标准。生态型经济枢纽六大支撑体系和海峡西岸经济区重要增长极基本形成。社会主义新农村建设明显推进，工业化与城镇化进程明显加快，对外开放明显扩大，可持续发展水平明显提升，和谐社会建设明显进步。

　　围绕实现上述目标，着重抓好六个方面工作：

　　1、着力推进社会主义新农村建设

　　始终把“三农”工作作为全部工作的重中之重，贯彻工业反哺农业、城市支持农村和多予少取放活的方针，加大对农业农村的投入，以“十镇百村”试点为示范，促进农业增效、农民增收、农村和谐。

　　拓宽农民增收渠道。巩固、完善和加强各项支农惠农政策。加快发展现代农业，在稳定粮食生产的基础上，深化农业结构调整，推进农业标准化生产，加强农产品质量安全监管，大力发展优势特色种养业和无公害农产品、绿色食品、有机食品，培育农产品品牌；加快农业科技进步，促进农业科技成果转化，鼓励农业科技进村入户；大力推进农业产业化，做大做强一批农业龙头企业和农民专业合作组织。积极推进与台湾的农业合作。扎实推进乡镇工业集中区建设，培育农村经济新增长点，拓展农民就业空间。加强农村人力资源开发，健全农民转移就业的支持保障体系，促进农村富余劳动力向二、三产业转移。

　　加强农村基础设施建设。加大农田水利建设力度，推进农村水、电、路、通信等设施建设，探索建立农村公路、水利设施管理维护机制。加强动植物疫病防控、防汛抗旱和林业“三防”等防灾减灾体系建设。推进农村“万村千乡”市场工程和农村现代化流通网络工程建设。继续实施农村“家园清洁行动”，推行“五清六改”，推进农村户用沼气工程建设，改善农村人居环境。

　　加快农村社会事业建设。加大农村义务教育投入，全面落实有关扶持政策，建立农村中小学校舍维修改造长效机制，加强农村寄宿制学校建设和农村现代远程教育。全面推行新型农村合作医疗制度，完善农村三级卫生服务网络，健全农村公共卫生和基本医疗服务体系。组织实施农村文化“百镇千村”建设工程和农村电影数字化放映“2131”工程，加强乡村文化、体育设施建设。

　　2、着力推进产业结构调整优化

　　坚持发展与调整、规模与效益相结合，努力形成科技含量高、资源消耗低、环境污染少、经济效益好、发展速度快的产业发展格局。

　　做大做强“10＋3”产业。滚动实施工业“三类项目”，加大对优势企业、龙头企业的扶持力度，加快培育一批拥有自主知识产权、主业突出、竞争力强的大企业、大集团，促进产业链延伸与产业配套，力争到2011年产值超亿元的工业企业达到200家，规模以上工业企业达到1800家，形成以大企业为主导，中小企业专业化分工、产业化协作、配套化发展的产业组织体系。加强工业园区建设，完善规划布局，继续抓好工业用地报批征用和标准厂房建设，健全基础设施和配套服务体系，提高园区集约化水平，努力培育若干个每平方公里产值达到或超过50亿元、100亿元的园区。落实“六个一”工作机制，努力把我市建成全国环保设备重要研发生产基地、南方重要的烟草生产加工基地和海峡西岸经济区重要的工程机械、运输机械生产基地、铜材料供应与加工基地、水泥建材基地、能源生产基地、钢铁生产基地、纺织服装生产基地和农产品加工生产基地。

　　切实转变经济增长方式。加快推进自主创新，继续组织实施一批重大科技项目，加强重点产业技术创新和企业技术中心建设，完善参加“6·18”项目成果交易会的常年对接机制，积极发展高新技术产业，建成市科技创业园，构建以企业为主体、市场为导向、产学研结合的区域技术创新体系。大力加强节能降耗，突出抓好高耗能行业和企业的节能，扎实推进年产3万吨以下小煤矿的关闭和单机5万千瓦及以下小火电机组的关停，促进资源综合利用和清洁生产，加快发展循环经济。继续实施“人才强市”战略，完善人才激励机制，培养和引进一批掌握核心技术的人才、复合型科技人才、管理型人才和高技能人才，增强人才和智力支撑。加大品牌培育力度，支持企业争创品牌，加强品牌保护。

　　大力发展服务业。推进旅游资源整合，打造红色旅游、客家旅游、生态旅游和乡村旅游精品，提高旅游接待能力和服务质量，加强市场营销，密切区域协作，把龙岩建成福建西部风情旅游区和海峡西岸经济区重要的生态旅游区。培育大型物流企业，加快发展电子商务、连锁经营和物流配送。优化住房供应结构，重点发展面向广大群众的普通商品住房，强化房地产市场监管，促进房地产业持续健康发展。加快发展交通运输、金融、保险、信息、商务、社区服务等服务业。

　　3、着力推进项目带动战略深入实施

　　坚持不懈地实施项目带动战略，深化“二三四五”项目工作思路，拓展项目工作载体，增强项目带动实效。

　　突出抓好重大项目。继续组织实施“三个一百”和重点项目建设计划，提高“两个比重”。加快推进龙工出口生产基地、龙升高档电梯、紫金年产20万吨铜冶炼、紫金山金铜矿联合开采、马坑铁矿新增500万吨/年采选工程、日产2500吨以上新型干法旋窑水泥生产线和新龙马中重型卡车等重大项目建设。扎实开展形式多样的项目竞赛活动，按照“三个决不能”的要求，生成、引进和实施一批符合政策导向、带动力强、经济社会效益好的大项目、好项目。重视抓好项目的前期工作，加大前期经费投入，加强向上沟通争取，促进更多的项目获批建设。

　　继续优化投资环境。建成龙长高速公路、永武高速公路和龙厦铁路，建设永春至永定（闽粤界）高速公路，加快推进漳州港至武平（闽赣界）高速公路、永安至漳州高速公路漳平段、赣龙铁路复线、长汀至永安至沙县铁路、上杭至梅州铁路、龙岩至建宁铁路等项目前期工作，拓展冠豸山机场的航线和业务，加快形成联结沿海、拓展腹地的现代化立体交通网络。积极推进漳平火电厂改扩建、坑口火电厂二期、龙岩500千伏输变电工程等项目建设，提高电力保障水平。进一步完善和落实促进投资与企业发展的有关政策，清理、缩减各种规费，加快推进融资和公共技术等服务平台建设，积极发展各类中介服务机构，营造良好发展环境。

　　进一步健全项目工作机制。完善项目开发机制，推进政府部门、企业、投资者和科研院校共同开发项目，分级建立健全项目储备库，为企业和投资者提供更加有效的项目推介、咨询和跟踪服务。健全项目服务机制，加强领导和部门的挂钩服务，帮助项目业主解决在项目报批、征地拆迁、施工材料组织等方面遇到的问题。改进项目管理机制，强化分级管理，落实奖惩措施。严格项目建设责任制，实现项目建设质量、进度、投资、安全、廉政“五同步”。

　　4、着力推进区域经济协调发展

　　坚持统筹兼顾、合理规划，强化区域互动融合，促进城乡同步、区域协调、全面发展。

　　增强中心城市带动功能。抓好龙岩经济开发区和龙州工业园区建设，壮大中心城市经济实力。继续滚动实施一批城建项目，优先发展公共交通，完善供水、供电、供气等系统，推进城市污水、垃圾处理产业化，加快发展教育、文化、医疗等公共事业和酒店、商务等服务业，增强中心城市综合服务功能。抓好城市规划编制，注重城市特色，提高城市品位。按照“市区一体、责权统一，建管并举、疏堵结合”的原则，健全城市管理长效机制，强化城市综合执法，坚决制止和查处违法违章建设，切实提高城市管理水平。

　　促进县域经济加快发展。进一步处理好条块关系，推进简政放权，健全有利于县域经济发展的体制机制。强化财政、信贷、科技、人才等的支持，促进各县（市、区）大力发展特色经济和园区经济，加快工业化、城镇化进程。加大对欠发达地区基础设施、产业发展、社会事业等的扶持力度，缩小县域发展差距。完善城镇体系规划，继续抓好小城镇和新村规划建设。

　　提升区域协作水平。加强与厦门的山海协作、对口帮扶和厦泉漳龙城市联盟建设，扎实推进基础设施共建共享、产业协作配套、市场相互开放和生态环境协同保护，山海联手壮大闽西南一翼。继续深化闽西南5市、闽粤赣边和闽粤赣13市的区域经济技术协作。

　　5、着力推进改革开放

　　大力推进体制改革和机制创新，进一步扩大对外开放，增强又好又快发展的动力和活力。

　　深化体制改革。全面推进农村综合改革，进一步创新农村工作机制。妥善处理国有企业改革历史遗留问题，加强已改制企业的规范管理。完善国有资产监管体制，建立国有资本收益收缴制度，健全国有资本经营激励约束机制。深化投资体制改革，推进企业投资项目核准制和备案制，进一步规范政府投资管理行为。加快财政体制改革，深化部门预算、国库集中支付、政府采购、“收支两条线”管理制度改革，加强税收征管。继续做好化解乡村债务工作。完善卫生监督体制，健全县（市、区）疾病预防控制体系。继续推进文化、教育体制改革。巩固普通公路征费体制改革成果。

　　扩大对外开放。积极有效利用外资，按照产业政策加大招商力度，争取在引导外资投向重点产业、高新技术产业等方面有新突破，在吸引外资来岩建立生产基地、研发中心、营销中心、地区总部等方面有新成效。主动承接台湾机械、轻纺、电子等产业转移，密切龙台旅游协作。积极参与重大经贸招商活动，做大“11&#8226;18”中国龙岩投资项目洽谈会平台。健全签约项目的跟踪推进和协调服务机制，促进项目报批、到资、落地、建设。完善鼓励出口的扶持政策和服务措施，优化出口结构，扩大出口规模。

　　支持非公有制经济发展。认真落实各项政策措施，改进对非公有制企业的服务，推进社会资本与重点产业、基础设施、社会事业和服务业的有效对接，鼓励外出人员回乡创业。支持有条件的企业上市。依法保护非公有制企业的合法权益。加强对非公有制企业的引导和管理，促进企业依法经营。

　　6、着力推进和谐社会建设

　　坚持以人为本，注重社会公平，把解决民生问题摆在更加突出的位置，促进社会发展和可持续发展，建设和谐龙岩。

　　发展和谐的社会事业。坚持教育优先发展，扎实推进“双高普九”，促进普通高中教育与中等职业教育协调发展，提高高等教育办学质量，支持和规范民办教育发展，加强教师队伍建设和教育资源整合。加快发展文化事业和文化产业，进一步打造红色文化、客家文化、河洛文化品牌，加强历史文化名城、名镇、名村和非物质文化遗产保护与开发，繁荣广播电视，维护良好的文化市场秩序。加快建设以社区为基础的新型城市卫生服务体系，做好重大传染病防治工作，推进城乡医疗救助工作，继续对重点优抚对象和革命“五老”人员实施医疗补助。强化食品、药品监管。发展体育事业，推进全民健身。落实人口与计生工作责任制，稳定低生育水平，改善人口结构，提高出生人口素质。继续做好就业再就业工作，健全建设领域工程款和农民工工资支付保障机制，更好地解决城市外来务工人员子女教育等问题，发展和谐劳动关系。加强社会保障体系和城乡社会救助体系建设，加大扶贫开发力度，继续实施“造福工程”。加快发展慈善事业。

　　保护和谐的生态环境。加大污染治理力度，力争2008年、确保2009年完成中心城市13家水泥污染企业关闭，扎实推进除中心城市以外其它污染企业的关闭工作，确保2010年年底前全部关闭。继续抓好九龙江、汀江、闽江等重点流域水环境污染综合整治，控制农村面源污染，推进垃圾、污水、噪音等的综合治理。严格执行建设项目环保“三同时”制度，加强环境监测与执法，切实控制和削减排污总量。重视资源节约，整顿和规范矿产资源开发秩序，实行最严格的耕地保护制度，加强城乡饮用水源保护。强化“青山挂白”、重点水土流失区治理和地质灾害防治，抓好植树造林和生态公益林管护。

　　营造和谐的社会环境。扎实推进“平安龙岩”建设与社会治安综合治理，加强应急管理体系建设和应急管理工作，维护社会安定稳定。健全安全生产管理长效机制，深化安全生产专项整治，促进安全生产状况持续好转。深入整顿和规范市场经济秩序，加快社会信用体系建设。加强社区建设，改善村（居）委会办公条件，完善基层管理和服务网络。推进和谐创建活动与群众性精神文明创建活动有机结合，营造和谐文明的社会氛围。巩固军政军民团结，加强“双拥”、国防动员、民兵预备役、武警消防和人民防空工作。继续抓好统计、民族宗教、外事侨务、老区、信访、气象、水文、普法、档案、地方志、老年人、妇女儿童、残疾人等工作。

　　落实“四个重在”　认真做好2007年工作

　　各位代表，2007年全市经济社会发展的主要预期目标是：地区生产总值增长11%、总量超过500亿元，全社会固定资产投资增长20%，全部工业总产值增长19.5%，规模以上工业总产值增长23.5%，财政总收入、地方级财政收入分别增长13%以上，实际利用外资增长50%，内联引资实际利用市外资金增长50%，外贸出口增长30%，社会消费品零售总额增长12%，城镇居民人均可支配收入增长9%，农民人均纯收入增长5.5%，万元GDP能耗下降5%，城镇登记失业率控制在4.5%以内，居民消费价格指数上涨幅度控制在3%以内，人口自然增长率控制在9‰以内，规模以上工业经济效益综合指数保持全省第一，工业用地平均投资额有较大幅度增长。实际工作中力争完成得更好更快些。按照“四个重在”的要求，主要从四个方面抓好落实：

　　1、重在持续，强化产业支撑，保持良好发展势头。

　　积极发展现代农业。以发展现代农业为重点，扎实推进社会主义新农村建设。粮食播种面积稳定在285万亩以上，大力发展畜牧、蔬菜、林竹、果茶、烟草、花卉等优势产业和红心地瓜干、河田鸡、连城白鸭、咸酥花生、黄兔等特色产品。健全基层农业技术推广和服务体系，发展高产、优质、生态农业。重点扶持肉制品、果蔬制品、竹制品加工企业。加快发展首批15个乡镇工业集中区。抓好漳平（永福）台湾农民创业园等7个龙台农业合作示范区和台湾优良品种引进繁育中心、农产品质量安全检测中心建设。完成农村劳动力职业技能培训4.5万人，争取新增转移农村富余劳动力6.3万人。

　　大力发展工业经济。加快发展“10+3”产业，推进工业“九大基地”建设。确保180个工业重点新增长点项目实现新增产值75亿元以上，90个工业重点投资项目完成投资52亿元，100个重点技术创新、新产品开发和电子信息应用项目顺利实施。精心组织实施“百家千户”培育工程，力争年内产值超亿元工业企业达到100家，规模以上工业企业达到1000家。支持福建煤电、马坑矿业等企业上市。加快工业园区建设，继续做好万亩工业用地报批征用和标准厂房建设工作，鼓励发展“飞地工业”。

　　加快发展服务业。抓好冠豸山、客家土楼、红色旅游区和乡村旅游区建设，积极开展创建国家4A级旅游区、中国优秀旅游县、全国农业旅游示范点等活动，办好市第五届旅游节，力争全年接待游客和实现旅游总收入分别增长17%和15%。加快闽西交易城二期、闽西粮油饲料城二三期、闽西现代物流园区、汽车及配件专业市场等物流项目建设。加大普通商品住宅和经济适用房及廉租房建设力度，规范物业管理。组织好第三届工业企业银企洽谈会，落实对金融机构新增贷款的奖励政策。

　　2、重在提升，不断提高经济增长的质量和效益，增强综合竞争力。

　　提高对外开放水平。在巩固港澳台和沿海发达地区等招商区域的基础上，进一步扩大对东南亚、欧美发达国家和长三角、珠三角等地区的招商。用好“5·18”、“6·18”、“9·8”等大型招商平台，办好建市10周年庆祝活动和第四届“11&#8226;18”中国龙岩投资项目洽谈会。加强产业招商和园区招商。切实提高签约项目的履约率。落实出口退税和外贸出口奖励政策，完善“大通关”机制，扩大外贸出口。

　　增强自主创新能力。加快市科技创业园和面向重点产业的8个公共技术服务平台建设，争取新建市级以上企业技术中心5家。认真组织参加第五届“6·18”项目成果交易会，促进项目成果常年对接和交易。完善政府推动与社会支持相结合、学校教育与企业培养相结合的创新型人才培养体系，加强自主创新人才队伍建设。

　　提升可持续发展能力。突出抓好冶金、煤炭、电力、化工、建材等行业的节能工作，建立10家循环经济示范企业，新增工业废弃物综合利用企业2—3家。全面推进重点流域水环境污染综合整治，其中九龙江流域基本完成养殖业治理任务，汀江、闽江流域完成50头以上规模养猪场的治理任务。关闭中心城市第二批3家水泥污染企业，强力推动除中心城市以外其它污染企业的关闭。启动龙岩化工厂搬迁。加强国土资源规划管理，提高科学开发利用水平。

　　3、重在运作，加快集聚各种资源要素，拓展发展空间。

　　深化项目带动。继续组织实施“三个一百”项目建设，安排新增长点项目193个，年新增产值75.7亿元；投资项目288个，年度投资143.2亿元；储备项目163个，总投资553.8亿元。安排重点项目110个，年度投资101.3亿元，其中，在建重点项目46个，年度投资76.2亿元；预备新开工项目50个，年度投资25.1亿元；前期工作项目14个。确保龙长高速公路年内建成通车，永武高速公路和龙厦铁路全线开工建设；马坑铁矿二期技改、漳平红狮一期等项目建成投产；龙工出口生产基地、龙升高档电梯、龙岩500千伏输变电工程等项目取得明显进展。深入开展“双百”项目竞赛活动。

　　推进改革创新。加快乡镇机构、农村义务教育管理体制和县乡财政体制改革，推进粮食流通体制、集体林权制度、小型水利设施产权制度、征地制度和农村金融改革。加强乡镇“三农”服务中心和“农民之家”建设，完善农村“六大员”服务机制。争取全面完成市属国有企业改制。加快市属经营性国有资产授权监管步伐。实施第二批市直行政事业单位和县级财政国库集中支付改革试点，制定并实施新一轮市对新罗区和龙岩经济开发区的财政预算管理体制。推行政府投资项目代建制和经评审的最低价中标办法。

　　加强联动互动。完成中心城市总规修编；建成2003—2007年百个城建项目，抓好今年39项城市道路、污水管网和市政改造项目建设；加快建设中心城市第二水源，开工建设博物馆、会议中心；进一步理顺城市管理体制，强化以交通、卫生、市场秩序、小区管理、夜景灯光为重点的城市综合管理，坚决制止和查处违法违章建设。加强对县域经济发展的指导与服务，激发县域发展活力。扎实推进区域经济技术协作、城市联盟和山海协作、对口帮扶。

　　4、重在实效，突出以人为本，促进社会和谐文明。

　　切实改善民生。落实就业再就业扶持政策，完善就业援助、劳务派遣机制，帮助城镇“零就业家庭”、农村贫困家庭和就业困难人员就业，创建和谐劳动关系企业和工业园区。扩大社会保险覆盖面，全面实施农民房屋保险，推进农业保险，实施农村部分离任村（居）主干养老补助，积极探索建立适合被征地农民和农民工特点的社会保障制度。做好城乡低保工作，提高农村居民最低生活保障标准，推广城乡医疗救助制度。落实大中型水库移民后期扶持政策。实施25项为民办实事项目。

　　促进社会事业全面发展。完成30万平方米农村中小学D级危房改造，健全职业教育和培训网络，推进龙岩学院、闽西职业技术学院迎评前期工作。认真做好迎接联合国专家组来岩考察永定土楼申报“世遗”工作，启动古田会议旧址群维修保护三期工程，完成闽西革命历史博物馆展馆改版，精心组织纪念建军80周年等重大活动，改造完善20个乡镇综合文化站。完成10万用户有线电视数字化整体转换。加快实施乡镇卫生院“百院建设、千人培训”计划，选派百名医生帮扶百所乡镇卫生院，全面推行新型农村合作医疗制度；加快组建市级妇幼保健机构，大力发展社区卫生服务。加强体育设施的管理与开发，加快发展体育产业。有效治理出生人口性别比偏高问题，完善流动人口管理服务体系，健全利益导向机制，确保人口与计生工作持续健康发展。

　　营造安定稳定的社会环境。扎实推进“平安龙岩”创建活动，健全社会治安防控体系，推进“三基”工作，强化信访和矛盾纠纷调查排处，继续整治治安突出问题。健全应急管理机制，有效预防和处置突发公共事件。深入开展安全生产“双基”建设深化拓展年活动，坚决遏制重特大安全事故。

　　大力加强政府自身建设

　　进一步树立对人民高度负责的政府形象

　　各位代表，推进科学发展、构建和谐社会的新形势、新任务，对政府自身建设提出了新的更高要求，我们将始终坚持执政为民，进一步加强政府自身建设，提高执行力和公信力，努力建设行为规范、公正透明、勤政高效、清正廉洁的政府，扎实推进龙岩又好又快发展，以实际行动担负起人民赋予的重任。

　　全面推进依法行政，严格规范政府行为。认真贯彻《中华人民共和国各级人民代表大会常务委员会监督法》，自觉执行人大及其常委会的决议决定，接受人大及其常委会的法律监督、工作监督和政协的民主监督，认真办理人大代表建议和政协提案，虚心听取民主党派、工商联、无党派人士和各人民团体的意见。深入贯彻国务院《全面推进依法行政实施纲要》和省政府《实施意见》，严格依照法定权限和程序行使权力。把实行科学民主决策、推进依法行政、加强行政监督作为政府工作的三项基本原则。

　　加快转变政府职能，推进政府管理创新。把政府抓经济工作的主要精力放在为各类市场主体服务和创造良好发展环境上，进一步加强社会管理和公共服务。深化行政审批制度改革，继续减少和规范行政审批事项，加强已取消审批项目的后续监管，推进部门内部相对集中行政审批职能改革。健全市、县两级行政服务网络，加快县级行政服务中心建设，完善服务机制，推行电子政务，提高服务效率。深化机关效能建设和政府系统绩效评估工作，切实改进直接面对群众和企业的“第一道门槛”的服务。

　　深入开展反腐倡廉，维护社会正义正气。坚持“严”字当头，严格责任，严格管理，严明纪律。严格执行廉洁自律的各项规定，加强社会主义核心价值体系建设，筑牢拒腐防变的思想道德防线。推进公共资源市场化配置工作，健全工程建设、政府采购、国有产权转让、探（采）矿权转让、国有土地出让等公开招投标制度，堵塞管理漏洞，降低行政成本，从源头上预防和治理腐败。认真治理和坚决纠正损害群众利益的不正之风，扎实开展治理商业贿赂专项工作。强化新闻舆论监督和社会公众监督，加大廉政监察和审计监督力度，推进政务公开，严防权力滥用。

　　切实加强队伍建设，大力倡导优良作风。认真贯彻实施《中华人民共和国公务员法》，按照胡锦涛总书记大力倡导八个方面良好风气的要求，努力建设一支素质高、能力强、作风好的公务员队伍。认真组织学习现代经济知识、科技知识、管理知识和法律知识，提升驾驭市场经济的能力、推进自主创新的组织领导能力、管理社会的能力和依法办事的能力。坚持一具体就深入、一具体就突破、一具体就落实，在解决问题、推动工作、致力发展中有效作为。坚持顾全大局、令行禁止，发扬民主、团结共事，持续风正气顺、人和业兴的良好局面。进一步解决人民群众最关心、最直接、最现实的利益问题，多办顺应民意、化解民忧、为民谋利的实事。

　　各位代表，回首过去我们信心倍增，展望未来我们豪情满怀。让我们更加紧密地团结在以胡锦涛同志为总书记的党中央周围，高举邓小平理论和“三个代表”重要思想伟大旗帜，全面贯彻落实科学发展观，在市委的领导下，团结和依靠全市人民，振奋精神，求真务实，开拓进取，为又好又快地推进龙岩新一轮大发展，加快生态型经济枢纽和海峡西岸经济区重要增长极建设，发挥海西纵深推进的前锋作用，为龙岩更加美好的明天而努力奋斗！
